# Epigenetic regulator BMI1 promotes alveolar rhabdomyosarcoma proliferation and constitutes a novel therapeutic target

**DOI:** 10.1002/1878-0261.12914

**Published:** 2021-03-27

**Authors:** Cara E. Shields, Sindhu Potlapalli, Selma M. Cuya‐Smith, Sarah K. Chappell, Dongdong Chen, Daniel Martinez, Jennifer Pogoriler, Komal S. Rathi, Shiv A. Patel, Kristianne M. Oristian, Corinne M. Linardic, John M. Maris, Karmella A. Haynes, Robert W. Schnepp

**Affiliations:** ^1^ Aflac Cancer and Blood Disorders Center Department of Pediatrics Division of Pediatric Hematology, Oncology, and Bone Marrow Transplant Emory University School of Medicine Atlanta GA USA; ^2^ Winship Cancer Institute Emory University Atlanta GA USA; ^3^ Children’s Healthcare of Atlanta Atlanta GA USA; ^4^ Department of Pediatrics Perelman School of Medicine University of Pennsylvania Philadelphia PA USA; ^5^ Department of Pathology and Laboratory Medicine Children's Hospital of Philadelphia University of Pennsylvania Philadelphia PA USA; ^6^ Division of Oncology and Center for Childhood Cancer Research Children’s Hospital of Philadelphia Philadelphia PA USA; ^7^ Department of Pediatrics Duke University Medical Center Durham NC USA; ^8^ Department of Pharmacology & Cancer Biology Duke University Medical Center Durham NC USA; ^9^ Wallace H. Coulter Department of Biomedical Engineering Emory University Atlanta GA USA

**Keywords:** BMI1, epigenetics, Hippo, pediatric cancer, rhabdomyosarcoma

## Abstract

Rhabdomyosarcoma (RMS) is an aggressive pediatric soft tissue sarcoma. There are two main subtypes of RMS, alveolar rhabdomyosarcoma (ARMS) and embryonal rhabdomyosarcoma. ARMS typically encompasses fusion‐positive rhabdomyosarcoma, which expresses either PAX3‐FOXO1 or PAX7‐FOXO1 fusion proteins. There are no targeted therapies for ARMS; however, recent studies have begun to illustrate the cooperation between epigenetic proteins and the PAX3‐FOXO1 fusion, indicating that epigenetic proteins may serve as targets in ARMS. Here, we investigate the contribution of BMI1, given the established role of this epigenetic regulator in sustaining aggression in cancer. We determined that *BMI1* is expressed across ARMS tumors, patient‐derived xenografts, and cell lines. We depleted BMI1 using RNAi and inhibitors (PTC‐209 and PTC‐028) and found that this leads to a decrease in cell growth/increase in apoptosis *in vitro,* and delays tumor growth *in vivo*. Our data suggest that BMI1 inhibition activates the Hippo pathway via phosphorylation of LATS1/2 and subsequent reduction in YAP levels and YAP/TAZ target genes. These results identify BMI1 as a potential therapeutic vulnerability in ARMS and warrant further investigation of BMI1 in ARMS and other sarcomas.

## Graphical abstract

Treatment of alveolar rhabdomyosarcoma (ARMS) remains a major therapeutic challenge in pediatric oncology. Here, we demonstrate that genetic and pharmacologic inhibition of BMI1 reduces ARMS viability. We show that BMI1 inhibits the tumor suppressive Hippo pathway and, conversely, that BMI1 disruption upregulates Hippo signaling. Collectively, these findings provide an initial framework for targeting BMI1 in ARMS and additional sarcomas.

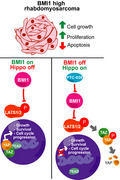

## 1 INTRODUCTION

Rhabdomyosarcoma (RMS) is a tumor of developing skeletal myoblast‐like cells that primarily afflicts children [1]. There are two major subtypes of pediatric rhabdomyosarcoma, alveolar, and embryonal, which are named based upon their histologic appearance. Approximately 80% of alveolar rhabdomyosarcomas (ARMS) are characterized by the presence of either PAX3‐FOXO1 or PAX7‐FOXO1 fusion proteins and are thus termed fusion‐positive; embryonal rhabdomyosarcomas (ERMS) lack these fusions and are termed fusion‐negative [1, 2]. ARMS is more aggressive and has a worse outcome compared with ERMS. The prognosis is even more dire for ARMS patients with metastatic dissemination, who have survival rates of only 30% [3, 4]. Currently, the standard of care is multimodal and intensive, consisting of multiagent chemotherapy, radiation, and surgery [5, 6]. Given the substantial morbidity and mortality of ARMS, there is a need for new, translatable treatment options.

While the PAX‐FOXO1 fusion proteins are pathognomonic for this disease, these proteins remain challenging drug targets [1, 7, 8, 9]. To date, efforts to pharmacologically inhibit PAX‐FOXO1 have not yielded robust clinical results [7]. Moreover, a recent study has shown that PAX3‐FOXO1 is necessary for the initiation/maintenance of ARMS but may not be required for recurrence, suggesting that the targeting of diverse oncogenic networks may be necessary to optimize the treatment of this cancer [8, 10]. The interaction of PAX‐FOXO1 fusions with the epigenome has garnered increasing attention [10, 11, 12]. PAX3‐FOXO1‐mediated gene regulation requires BRD4 at superenhancers, revealing a novel epigenetic vulnerability in ARMS [11]. Further, the fusion protein also requires the chromatin remodeling activity of CHD4 to activate a subset of its target genes [13]. Epigenetic regulation may also act upstream of PAX3‐FOXO1. Histone deacetylases control the expression of SMARCA4, a chromatin remodeler, which subsequently allows expression of *miR‐27a,* which in turn decreases *PAX3‐FOXO1* mRNA stabilization [14]. These studies provide evidence for a significant relationship between the epigenome and the tumorigenicity of ARMS, and suggest that druggable epigenetic regulators other than PAX3‐FOXO1 remain to be discovered.

Inspired by the studies highlighted above, we carried out a search for druggable epigenetic proteins involved in ARMS that may represent dependencies. The polycomb group proteins are epigenetic complexes traditionally associated with gene repression by chromatin compaction [15]. These complexes are known regulators of pluripotency, stem cell renewal, and epigenetic memory, and have been studied extensively across species and various human diseases [16]. They consist of polycomb repressive complexes 1 and 2 (PRC1/2) which control monoubiquitination of H2AK119 and trimethylation of H3K27, respectively [15, 17, 18, 19]. Dysregulation of PRC1/2 protein members are implicated in tumor initiation and progression in many adult cancers but remain relatively understudied in pediatric cancers [20]. High levels of H2AK119Ub and H3K27me3 across the genome in many cancers are associated with worse outcomes [18, 20], possibly due to the repression of tumor suppressor genes such as *CDKN2A*, which has a significant role in controlling the cell cycle [21]. Specifically in ARMS, PRC2 members such as EZH2 have been analyzed and found to promote survival [22]. Thus, we hypothesized that a member of PRC1, B lymphoma Mo‐MLV insertion region 1 (BMI1), also known as polycomb group factor 4 (PCGF4), would be a viable epigenetic target in ARMS. BMI1 has no enzymatic activity itself but is a required component of PRC1 and is a known oncogene in numerous adult cancers including hematological malignancies, breast cancer, ovarian cancer, and more [15, 20, 23, 24, 25, 26]. BMI1 has also been studied in pediatric cancers such as medulloblastoma and Ewing sarcoma, but its possible role in RMS has not yet been identified [27, 28, 29]. Additionally, BMI1 has been found to promote self‐renewal in skeletal muscle and was also one of the components, along with TERT and PAX3‐FOXO1, used to transform normal human myoblasts into a cell culture model of ARMS [30, 31]. In these studies, we identify BMI1 as a novel therapeutic liability in ARMS.

## 2 Materials and methods

### 2.1 *In silico* data

RNA‐sequencing data (dbGaP accession # phs001437) of six RMS patient‐derived xenograft (PDX) models and cell lines are from the NCI Pediatric Preclinical Testing Consortium (PPTC) [32, 33]. PDX samples were de‐identified and have no patient data associated with them [32, 33]. RNA‐sequencing data were processed using the STAR alignment tool and subsequently normalized using the RSEM package based upon the hg38 reference genome and the GENCODE v23 gene annotation. Gene expression values were quantified as fragments per kilobase per million mapped reads (FPKM).

### 2.2 Cell culture

Rhabdomyosarcoma cell lines (Rh30 and Rh41) were obtained from the Children’s Hospital of Philadelphia (courtesy of Dr. Margaret Chou) as well as from the Children’s Oncology Group (Rh28 and CW9019). The Emory Genomics Core authenticated cell lines for use and *Mycoplasma* testing was performed every 3–6 months using the *Mycoplasma* test kit (PromoCell, PK‐CA91‐1024). Cells were cultured in a humidified incubator at 37 °C with 5% CO_2_. Rh30 and CW9019 were passaged regularly in DMEM (Corning, Bedford, MA, USA), and Rh28 and Rh41 were passaged in RPMI 1640 (Corning). Media was supplemented with 10% FBS (Corning) and 1% l‐glutamine (Gemini, West Sacramento, CA, USA). No antibiotics or antimycotics were added to the media.

### 2.3 Plasmids, lentiviral preparation, and transduction

BMI1 shRNA plasmids were purchased from Sigma (St. Louis, MO, USA) (pLKO.1). The catalog numbers are shBMI1‐2: TRCN0000020156 and shBMI1‐4: TRCN0000218780. YAP‐overexpression plasmids pGAMA‐Empty (Addgene plasmid #74755) and pGAMA‐YAP (Addgene plasmid #74942) were kind gifts from Jenny Shim in Kelly Goldsmith’s laboratory at Emory University. Generation of infectious lentiviral particles and subsequent cell transduction was performed as previously described [34] with the following key conditions: FuGENE 6 (Promega, Madison, WI, USA) was used to transfect select plasmids, with pMD2. G (VSV‐G plasmid) and psPAX2 (packaging plasmid), into HEK293T cells. Viral supernatant was collected 2–3 days after transfection, filtered with a 0.45‐µm nitrocellulose membrane, supplemented with 8 µg/mL polybrene (Sigma), and used for transduction of one million cells seeded into 10‐cm plates. Fresh media was added 6 h post‐transduction, and the media was replaced again the next day. Two days later, puromycin was added to select for transgenic cells.

### 2.4 siRNA transfection

Cells were plated at 200 000 cells per well in a 6‐well plate. The following day, cells were transfected using DharmaFECT 1 (Horizon Discovery, Cambridge, UK) and 25 nm of an siRNA ON‐TARGETplus SMARTpool (Horizon Discovery) or ON‐TARGETplus Nontargeting Control Pool (Horizon Discovery). Horizon Discovery catalog numbers for each siRNA pool used in this study are as follows: ON‐TARGETplus Nontargeting Control Pool (D‐001810‐10‐20), BMI1 (L‐005230‐01‐0010), LATS1 (L‐004632‐00‐0010), and LATS2 (L‐003865‐00‐0010). Cells were harvested for analysis 72 h post‐transfection.

### 2.5 Real‐time PCR and western blots

RNA was isolated from cells using the RNeasy Mini Kit (QIAGEN, Hilden, Germany) and Real‐Time PCR (RT–PCR) analysis performed as previously described [34]. For western blots, cell samples were lysed in RIPA (Boston BioProducts, Ashland, MA, USA) containing cOmplete Protease Inhibitor Cocktail (Roche, Basel, Switzerland) and PMSF (Cell Signaling Technology, Danvers, MA, USA) then sonicated. Protein concentrations were determined using the Bradford assay (Bio‐Rad, Hercules, CA, USA), and samples (20 μg protein) run on SDS/PAGE Bis–Tris 4–12% gels (Life Technologies, Carlsbad, CA, USA). Lambda protein phosphatase (New England Biolabs, Ipswich, MA, USA) was used per manufacturer’s instructions on select cellular lysates. The gels were transferred to nitrocellulose membranes and membranes blocked in 5% Blotting‐Grade Blocker (Bio‐Rad) in Tris‐Buffered Saline with 1% Tween‐20 (Cell Signaling Technology). The blots were incubated with primary antibodies in 5% BSA (Jackson Laboratory, Bar Harbor, ME, USA) overnight at 4 °C. The secondary antibodies used were IRDye 800CW/680RD anti‐Rabbit or anti‐Mouse (Li‐COR Biosciences, Lincoln, NE, USA) at 1 : 50 000 and 1 : 5000, respectively. Whole blots were scanned using the Li‐COR Odyssey. The primary antibodies and dilutions are listed in Table S1. Any quantifications are presented as relative adjusted densities and were performed in ImageJ (Bethesda, MD, USA).

### 2.6 Cell growth assays

CellTiter‐Glo (Promega) and Caspase‐Glo (Promega) were used to assess viability of both shRNA/siRNA manipulated and drug‐treated cells. On day 0, 2000 cells/well were plated in a 96‐well plate and on day 1 treated with control or drug. To calculate IC50s, cells were treated with a 7‐log dose range of inhibitor (10^‐11 M^–10^‐5 M^). Cells proliferated for an additional 96 h before performing CellTiter‐Glo or Caspase‐Glo per the manufacturer’s instructions. IC50s were calculated by log‐transforming concentrations, fitting to a three‐parameter logistic nonlinear regression curve and finding the half‐maximal concentration [35].

For crystal violet colony formation assays, we plated 2000 cells/well in duplicate in 6‐well plates. We treated cells with drugged media and allowed cells to proliferate for 10 days prior to washing/fixing with 3.7% formaldehyde then staining with 0.0025% crystal violet. Plates were dried overnight and were imaged with a Nikon D3400.

### 2.7 Flow cytometry

On day 0, cells were seeded at 1 million cells/10 cm plate and PTC‐028 added on day 1. Cells were harvested after 24 h for BrdU‐APC/7‐AAD staining and 72 h for Annexin V‐FITC/PI staining. Staining was performed using Annexin V‐FITC/PI (BD Biosciences, Franklin Lakes, NJ, USA) or BrdU‐APC/7‐AAD (BD Biosciences) kits following manufacturer’s instructions. For Annexin V/PI staining, cell media containing dead cells in suspension was also collected. Samples were run within 1 h on a Cytoflex 96‐well plate loader, with 50 000–100 000 events collected per sample. Compensation, gating, and analyses were performed in FlowJo.

### 2.8 *In vivo* xenograft model

Heterozygous nude mice (Crl:NU(NCr)‐*Foxn1^nu^/^+^*) between 5 and 6 weeks old (Charles River, Wilmington, MA, USA) were housed in sterile cages at the Health Sciences Research Building Animal Facility at Emory University. Mice acclimated to their new environment for 1 week after being received and were maintained in 12‐hr day/night cycles. All experimental procedures were Emory IACUC approved. 2 million Rh30 cells were mixed 1 : 1 with Matrigel (Corning) and subcutaneously injected into the right flank of each mouse. As previously described, treatments began when tumors were equal to or greater than 100 mm^3^ [36, 37]. The mice were tagged and randomly separated into 2 groups: vehicle (*n* = 10) and PTC‐028 (*n* = 10). Mice received vehicle (0.5% HPMC, 1% Tween‐80) or 15 mg/kg PTC‐028 twice weekly by oral gavage [36, 37]. Weights and tumor sizes were measured three times weekly. Tumor volumes were calculated by using an ellipsoid volume formula: π/6 × L × W × H [38]. In accordance with the IACUC protocol, mice were sacrificed when tumors reached a volume greater than or equal to 1500 mm^3^. Collected tumors were removed postmortem and snap‐frozen in liquid nitrogen for immunoblotting or formalin fixed and paraffin embedded for immunohistochemistry.

### 2.9 Immunohistochemistry

Both a representative tumor array of pediatric solid tumors (duplicate punches) and an additional array comprising 41 normal pediatric tissues/organs (duplicate punches) were constructed at the Children’s Hospital of Philadelphia from 2005–2012. As de‐identified clinical materials were utilized to create arrays, the arrays were created under an IRB exemption (IRB‐13‐010191) [39]. BMI1 antibody (Cell Signaling Technology) was used to stain formalin‐fixed paraffin‐embedded tissue slides. Staining was performed on a Bond Rx automated staining system (Leica Biosystems, Wetzlar, Germany). The Bond Refine polymer staining kit (Leica Biosystems) was used. The standard protocol was followed apart from the primary antibody incubation which was extended to 1 h at room temperature, and the postprimary step was excluded [35]. BMI1 antibody was used at a 1 : 200 dilution, and antigen retrieval was performed with E1 (Leica Biosystems) retrieval solution for 20 min. Slides were rinsed, dehydrated through a series of ascending concentrations of ethanol and xylene, and then, coverslips were added. Stained slides were then digitally scanned at 20× magnification on an Aperio CS‐O slide scanner (Leica Biosystems). Tumor microarrays were scored by a pediatric pathologist (JP) for the most prominent intensity of nuclear staining (score 0–3 with 1 representing weak/equivocal, 2 moderate, and 3 strong positive staining) as well as for percentage of tumor nuclei staining [39]. An overall score was obtained by multiplying intensity by percentage of tumor cells staining. Both cores for each of the two cores per tumor were averaged for the final score.

### 2.10 Statistical analyses

Data analyses were performed in graphpad prism 8 (San Diego, CA, USA). Statistical significance was determined using an unpaired Student two‐tailed *t*‐test for two groups. Groups of three or more were analyzed using an ANOVA. All assays were performed in duplicate unless otherwise stated and presented using mean and standard deviation. Survival curves were generated in Prism 8 using the Kaplan–Meier method [40].

## 3 RESULTS

### 3.1 BMI1 is highly expressed in rhabdomyosarcoma

To investigate BMI1 as a potential therapeutic vulnerability in ARMS, we sought to define its expression pattern in sarcomas, broadly considered. We first examined Oncomine and determined the expression of *BMI1* in both adult and pediatric sarcomas [41]. We noted that *BMI1* is robustly expressed in pediatric sarcomas, such as Ewing sarcoma and osteosarcoma, as well as in adult subtypes, including leiomyosarcoma and chondrosarcoma (Fig. S1A, B) [28, 41, 42].

**Fig. 1:**
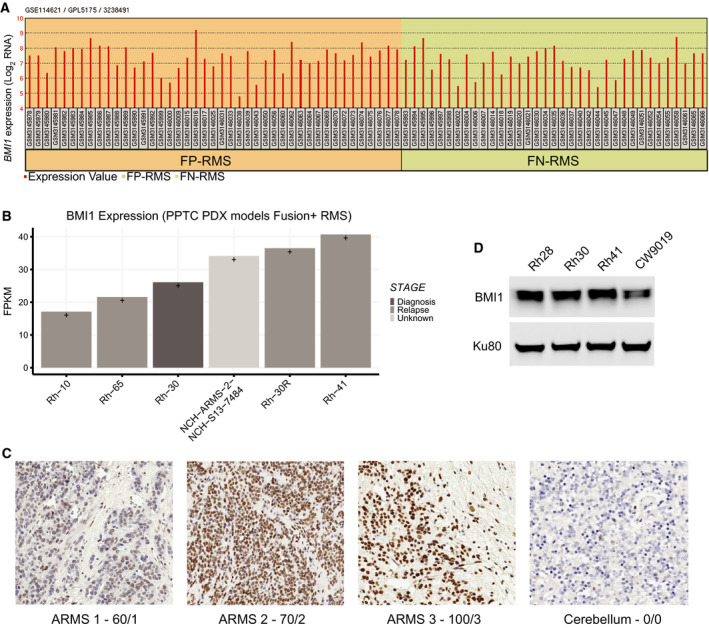
BMI1 is highly expressed in rhabdomyosarcoma. (A) Barplot of *BMI1* gene expression (Log2 RNA signal intensity) from human exome array data across fusion‐positive RMS and fusion‐negative RMS patient tumor samples (GSE114621) [43]. (B) Boxplot of *BMI1* gene expression values from RNA‐sequencing data of ARMS PDX and cell line models (*n* = 6). *Y*‐axis represents fragments per kilobase of exon per million reads (FPKM) values. (C) Tumor microarray with three representative ARMS tumors from patients and a negative control normal pediatric cerebellum. BMI1 is brown (DAB). The nuclear counterstain for BMI1‐negative cells is purple (hematoxylin). The first number refers to the percentage of tumor cell nuclei expressing BMI1, while the second number is the strength of the staining, which ranges from 0 (negative) to 3 (strong staining) [39]. (D) Western blot of ARMS cell lines Rh28, Rh30, Rh41, and CW9019 showing BMI1 protein expression with a Ku80 loading control.

We then focused on RMS. We began by interrogating available datasets and first examined human exon array data from both ARMS and ERMS patient tumor samples [43]. We observed that *BMI1* expression levels are expressed across both subtypes (Fig. 1A). To focus on ARMS specifically, we analyzed *BMI1* levels from RNA‐seq ARMS patient‐derived xenograft (PDX) from the Pediatric Preclinical Testing Consortium (PPTC) [44]. We found that *BMI1* mRNA levels are expressed in ARMS. (Fig. 1B). Furthermore, we probed the OncoGenomics database and found *BMI1* to be expressed in both ARMS and ERMS (Fig. S1C) [45]. Using immunohistochemistry, we stained a tumor microarray bearing ARMS patient samples and confirmed that BMI1 is expressed at the protein level (Fig. 1C), with normal pediatric cerebellum shown as a negative control.

Finally, we surveyed the expression of BMI1 across the ARMS cell lines Rh28, Rh30, Rh41, and CW9019 and find that BMI1 is expressed across all models (Fig. 1D). Notably, Rh28, Rh30, and Rh41 have the PAX3‐FOXO1 fusion, while CW9019 harbors the PAX7‐FOXO1 fusion [46].

### 3.2 Genetic knockdown of BMI1 leads to reduced cellular proliferation in ARMS cells

**Fig. 2:**
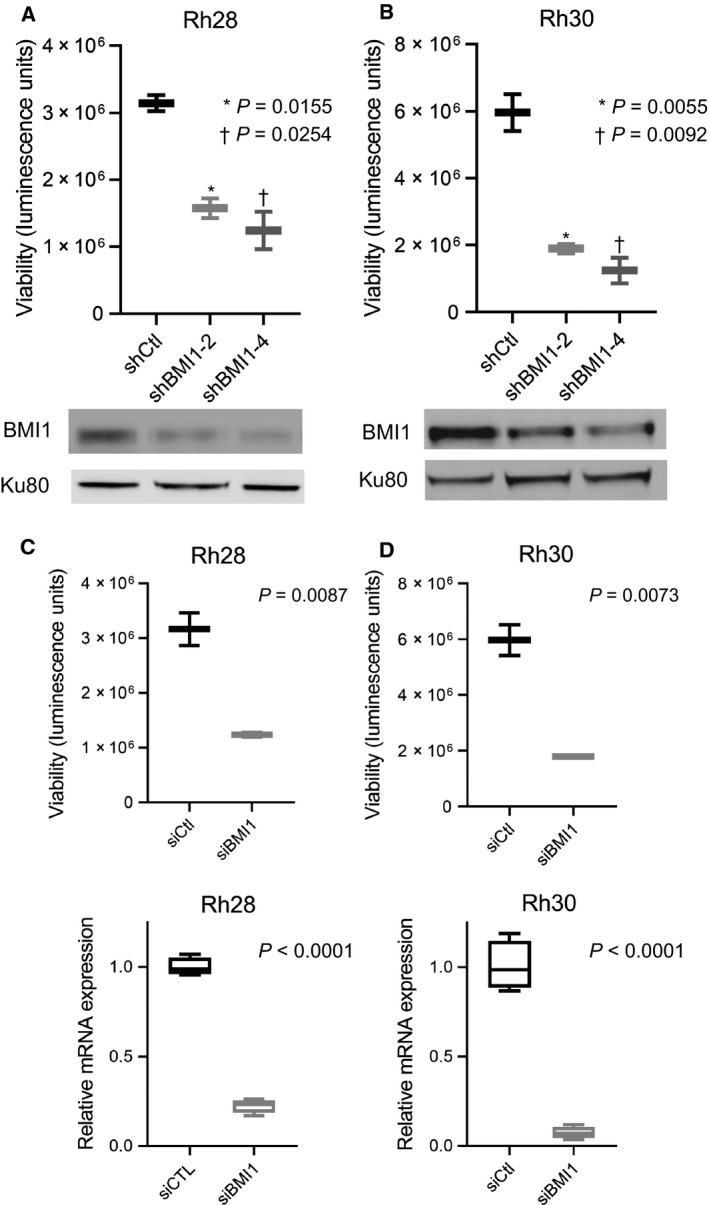
Genetic knockdown of BMI1 leads to reduced cellular proliferation in ARMS cells. (A) Rh28 (A) and Rh30 (B) cell lines were infected with control lentiviruses or lentiviruses expressing two independent shRNAs directed against BMI1. Cell proliferation in control and BMI1‐depleted cell lines as assessed by Cell‐TiterGlo. Western blotting of BMI1 and Ku80 in corresponding cell lines. (C,D) Rh28 (C) and Rh30 (D) cells were transfected with control siRNAs or pooled siRNAs directed against BMI1. Cell proliferation assessed by Cell‐TiterGlo, with corresponding siCtl and siBMI1 RT–PCR data depicted below. Standard deviation bars shown. Results are representative of at least three independent biological replicates (*n* = 3). Statistical significance was determined using an unpaired Student two‐tailed t‐test for two groups; *P*‐values are shown within the figure.

Our analyses demonstrate that BMI1 is highly expressed in both fusion‐positive and fusion‐negative rhabdomyosarcoma. Given the clinical aggression of ARMS, in subsequent investigations, we focused exclusively on this subtype. We chose two ARMS cell line models, Rh28 and Rh30, for genetic knockdown studies, as these models have been well‐studied and are readily transduced and transfected. First, we depleted BMI1 using two independent shRNAs directed against BMI1 and confirmed effective knockdown of BMI1 by western blot (Fig. 2A,B). We observed that BMI1 knockdown significantly reduces cell proliferation by CellTiter‐Glo, an assay which quantitates ATP to determine the number of viable cells present (Fig. 2A,B) [47]. To further validate these findings, we utilized pooled siRNAs (comprised of 4 different siRNAs directed against BMI1) to transiently deplete BMI1 and again demonstrated significantly decreased proliferation (Fig. 2C,D). Knockdown of *BMI1* was confirmed by RT–PCR (Fig. 2C,D). Thus, with both transient and longer‐term depletion of BMI1, we observed decreased proliferation.

### 3.3 Pharmacologic inhibition of BMI1 decreases cell proliferation *in vitro*

**Fig. 3:**
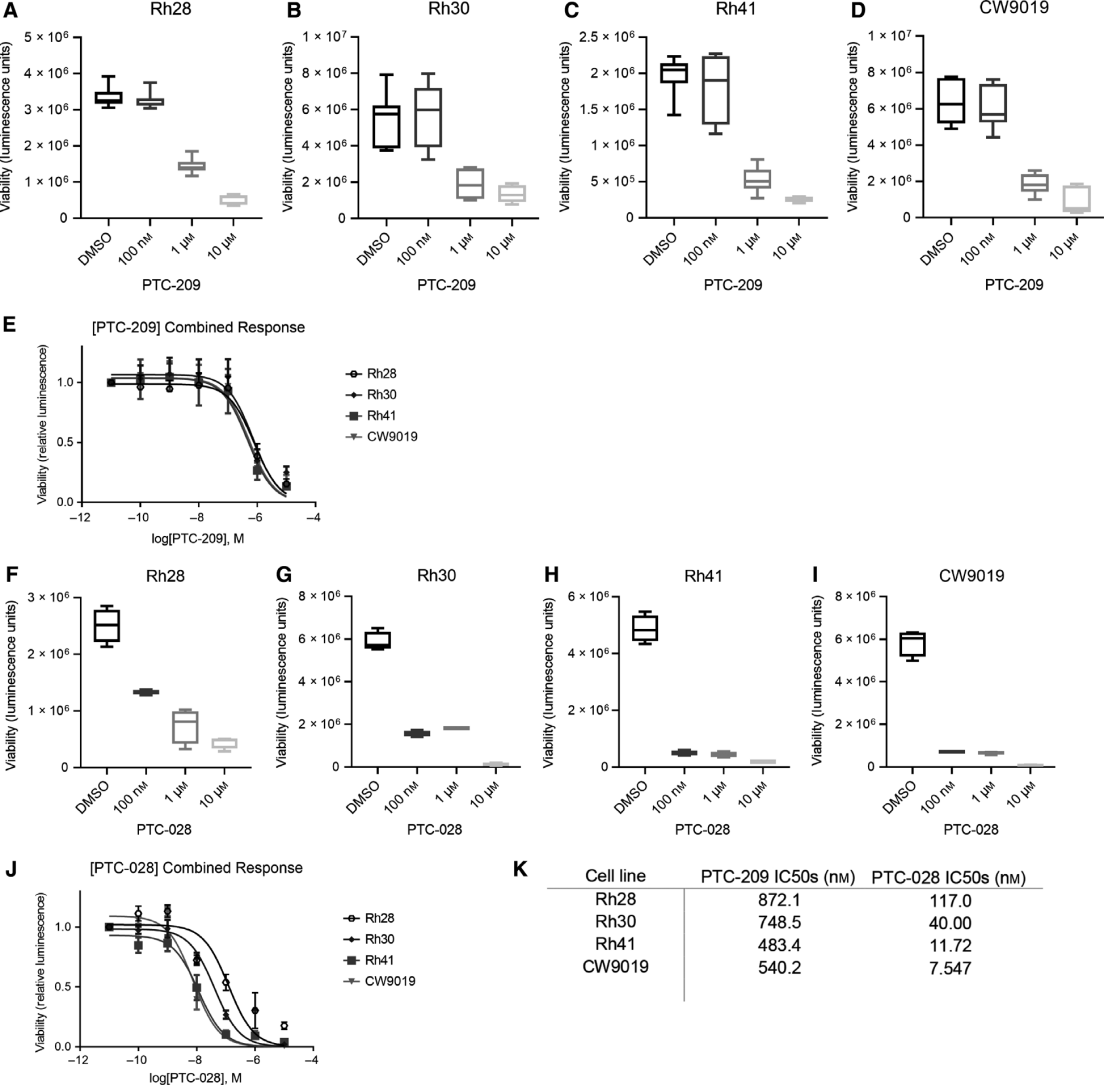
Pharmacologic inhibition of BMI1 decreases cell proliferation *in vitro*. (A‐D) Cell lines Rh28 (A), Rh30 (B), Rh41 (C), and CW9019 (D) were treated with a 7‐log dose range of PTC‐209. Graphs display cell viability measured with CellTiter‐Glo with varying concentrations of PTC‐209. (E) Dose–response curve of PTC‐209 ranging from 10^−11^ m to 10^−5^ m. (F‐I) Cell lines Rh28 (F), Rh30 (G), Rh41 (H), and CW9019 (I) were treated with a 7‐log dose range of PTC‐028. Graphs display cell viability measured with CellTiter‐Glo at varying concentrations of PTC‐028. (J) Dose–response curve of PTC‐028 ranging from 10^−11^ m to 10^−5^ m. (K) Table summarizing IC50 values of PTC‐209 and PTC‐028. Results are representative of at least three independent biological replicates (*n* = 3).

We assessed the effects of pharmacologic inhibition of BMI1 on ARMS. To do so, we initially employed PTC‐209, an inhibitor that reduces BMI1 protein levels and lowers PRC1 activity in cancer cells, with minimal effects in noncancerous cell line models [48]. In several aggressive cancer models, such as colorectal cancer and biliary tract cancer, PTC‐209 has been found to impair cell growth through promoting cell cycle arrest and causing cell death [48, 49]. Guided by previous studies, we treated 4 ARMS cell lines with PTC‐209 across a 7‐log dose range (10^‐11^ M–10^‐5^ M). Treatment with PTC‐209 significantly decreases cell proliferation (Fig. 3A–D) in all 4 cell lines, with IC50s ranging from 483 nm to 872 nm (Fig. 3K). Protein levels of BMI1 were also reduced with PTC‐209 treatment (Fig. S2A).

Next, we assessed the impact of a second‐generation BMI1 inhibitor, PTC‐028, on ARMS proliferation. PTC‐028 inhibits BMI1 by a different method than PTC‐209, resulting in hyperphosphorylation of BMI1 and disrupting its function [36]. It is also orally bioavailable, allowing for preliminary investigation of BMI1 disruption in the *in vivo* setting; for these reasons, in subsequent studies we employed PTC‐028. Treatment with PTC‐028 similarly decreases cell proliferation (Fig. 3F–J) in all 4 cell lines, yielding decreased BMI1 protein levels (Fig. S2A). To test whether this was due to hyperphosphorylation of the BMI1 protein as previously reported [36], we treated Rh30 cells with PTC‐028 at 100 nm and 1 μm, then collected lysates after 12 h (Fig. S2B). Some lysates were treated with a Lambda (λ) protein phosphatase to remove phosphorylation bands and confirm BMI1 protein loss. We indeed found that BMI1 is being hyperphosphorylated after PTC‐028 addition at both 100 nm and 1 μm doses, and the BMI1 protein level decreases slightly at 1 μm treatment (Fig. S2B). Next, as expected, IC50s were lower for PTC‐028 than for PTC‐209, consistent with the greater potency of PTC‐028 (Fig. 3K). Additionally, brightfield microscopy and colony formation assays showed that viability is significantly diminished with 50 nm and 100 nm doses of PTC‐028 in Rh30 and CW9019 (Fig. S2C, D). Thus, our data indicate that two BMI1 inhibitors greatly decrease proliferation in ARMS cell line models, mimicking the effects we observed with genetic disruption of BMI1.

### 3.4 Targeting BMI1 decreases cell cycle progression and increases apoptosis in ARMS

We next aimed to define the mechanisms by which BMI1 promotes cell proliferation. Previous investigations have demonstrated that BMI1 influences cell cycle progression in part through repression of the *CDKN2A* (*p16‐INK4a*) locus [50], although this regulation is not observed in all contexts. BMI1 also possesses functions independent of *CDKN2A* repression, including the regulation of genes involved in differentiation and cell contact inhibition in Ewing sarcoma and androgen receptor expression in prostate cancer [28, 51].

**Fig. 4:**
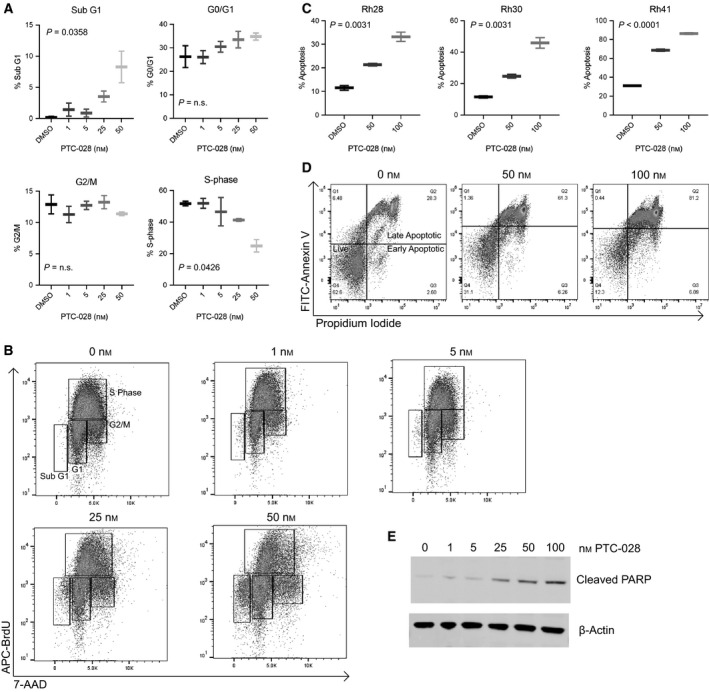
Targeting BMI1 decreases cell cycle progression and increases apoptosis in ARMS. (A) Graphs depict cell cycle distribution in the Rh30 cell line treated with PTC‐028 (0–50 nm for 24 h). (B) Representative cell cycle distribution from Rh30. BrdU is depicted on the y‐axis with 7‐AAD on the *x*‐axis. (C) Flow cytometry analysis of Annexin V/PI staining in Rh28, Rh30, and Rh41, with PTC‐028 treatment ranging from 0 to 100 nm for 72 h. (D) Representative example of flow cytometry data illustrating apoptosis with Annexin V (*y*‐axis) and propidium iodide (*x*‐axis). (E) Rh30 was treated with PTC‐028 for 72 h, with western blot depicting cleaved PARP and actin. Standard deviation bars depicted. Results are representative of at least three independent biological replicates (*n* = 3). Statistical significance was determined using an unpaired Student two‐tailed *t*‐test for two groups; p‐values are shown within the figure.

To investigate the influence of BMI1 on cell cycle progression, we treated Rh30 with PTC‐028 at doses below and near the IC50 of Rh30 and then performed BrdU/7‐AAD staining. We observed a ˜ 10% increase in the sub‐G1 population (*P* = 0.0358) and a 50% decrease in the percentage of cells in S phase (*P* = 0.0426) when the cells were treated with 50 nm of PTC‐028 for 24 h (Fig. 4A,B). Given the increase in the sub‐G1 population, we speculated that BMI1 additionally increases apoptosis *in vitro*. Therefore, we performed Annexin V/PI staining after 72 h of PTC‐028 treatment and observed a dose‐dependent increase (20–50% across cell lines, *P* < 0.05) in the percentage of apoptotic cells (Fig. 4C,D). To further verify the apoptotic phenotype, we probed for cleaved PARP and noted an increase in PARP cleavage with PTC‐028 addition (Fig. 4E). Additionally, to complement these data, we performed Caspase‐Glo analyses of shBMI1/siBMI1 Rh28 and Rh30 cell lines and discovered an increase (shBMI1: twofold to fourfold across cell lines, *P* < 0.05, siBMI1: threefold to fivefold, *P* < 0.05) in caspase 3/7 activity (Fig. S3A, B). We delved down further and analyzed apoptosis in siBMI1 transfected Rh28 and Rh30 cells by Annexin V/PI staining and again noted an increase (2.8‐ to fivefold, *P* < 0.05) in the apoptotic fractions (Fig. S3C). Together, these data confirm that pharmacologically targeting BMI1 impairs progression to S phase and results in apoptosis.

### 3.5 Single‐agent PTC‐028 treatment causes tumor growth delay *in vivo*

**Fig. 5:**
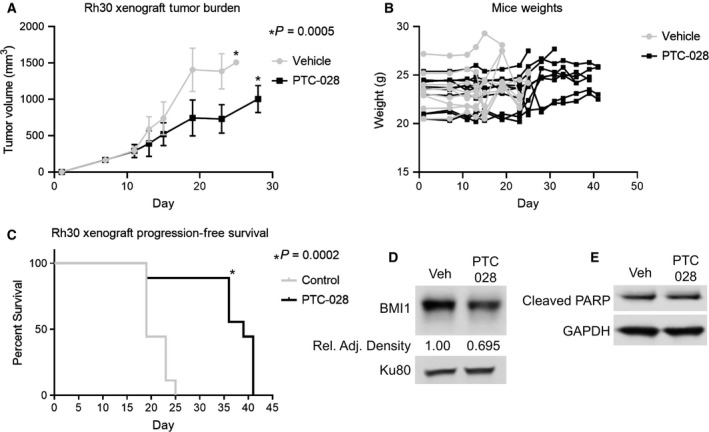
Single‐agent PTC‐028 treatment causes tumor growth delay *in vivo*. Rh30 xenografts were treated with vehicle or PTC‐028 (15 mg/kg 2x/weekly). (A) Response of tumor volumes to vehicle and PTC‐028. (B) Weight change from baseline on study arms. (C) Kaplan–Meier analyses for Rh30 xenografts. (D) Representative western blot of BMI1 and Ku80 in control‐ and PTC‐028‐treated tumors. (E) Western blot of cleaved PARP levels with GAPDH as a loading control. Results are representative of at least three independent biological replicates (*n* = 3). Statistical significance was determined using an unpaired Student two‐tailed *t*‐test for two groups; *P*‐values are shown within the figure.

To provide the initial foundation for targeting BMI1 in ARMS, we employed PTC‐028, which is orally bioavailable [36, 37]. Nude mice bearing Rh30 xenografts were treated with vehicle or PTC‐028 (15 mg/kg by oral gavage) daily, a dosing scheme guided by previous studies [36, 37]. As shown in Fig. 5A, treatment with PTC‐028 delays tumor growth in comparison with vehicle (Fig. 5A, *P* = 0.0005). The treatment was well‐tolerated, with no significant change in weights (Fig. 5B) and no signs of pain or distress in the mice observed. The vehicle group died by day 25, while the PTC‐028‐treated group survived until day 41 (Fig. 5C, *P* = 0.0002). The tumors were harvested and analyzed for BMI1 protein levels. By western blot, we noted that tumors in PTC‐028‐treated mice had an approximately 30% reduction in BMI1 levels in comparison with control. (Fig. 5D). Interestingly, however, in contrast to the *in vitro* setting, we noted no increase in cleaved PARP (Fig. 5E). Collectively, these results suggest that single‐agent treatment with PTC‐028 delays, though does not abrogate, the growth of an ARMS xenograft.

### 3.6 BMI1 negatively influences Hippo signaling

Given our findings demonstrating the positive influence of BMI1 on cell cycle progression, we first asked whether BMI1 inhibits *CDKN2A* expression in ARMS [52]. A canonical target of BMI1 is *CDKN2A*, and repression of *CDKN2A* controls cell cycle progression to S phase [50, 52]. We found that BMI1 inhibition by PTC‐028 treatment leads to a slight upregulation in CDKN2A protein levels in Rh30 (Fig. S4A).

We then undertook a candidate‐based approach to identify additional novel BMI1‐influenced signaling networks in ARMS. We focused on Hippo signaling for the following reasons: 1. BMI1 has been reported to interact with the Yes‐Associated Protein (YAP) in Ewing sarcoma, though whether this occurs in RMS is unclear [28], 2. PAX3‐FOXO1 has been found to suppress the Hippo pathway in ARMS [53], and 3. loss of Hippo signaling by Mst knockout was shown to accelerate ARMS tumorigenesis [54].

**Fig. 6:**
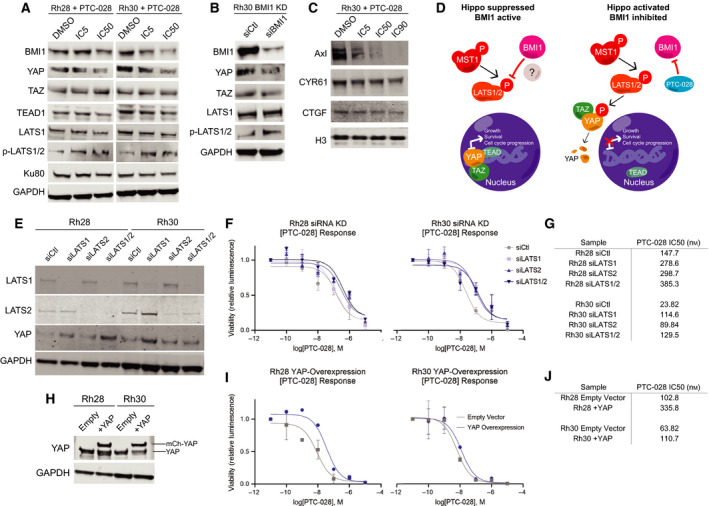
BMI1 negatively influences Hippo signaling. (A) Rh28 and Rh30 cells were treated with PTC‐028 at respective IC5 or IC50 concentrations for 72 h, with DMSO as a control. Western blot of BMI1 and Hippo pathway members YAP, TAZ, TEAD1, LATS1, p‐LATS1/2, and Ku80/GAPDH as loading controls. (B) Rh30 cells were transfected with an siRNA pool against BMI1, and western blot analyses were performed after 72 h. Western blot of BMI1 and Hippo pathway members YAP, TAZ, LATS1, p‐LATS1/2, and GAPDH as loading controls. (C) Rh30 cells were treated with PTC‐028 at respective IC5, IC50, or IC90 doses, with DMSO as a control. Western blot of AXL, CYR61, and CTGF with histone H3 as a loading control. (D) Model of BMI1 involvement in the Hippo pathway. In ARMS, BMI1 inhibits Hippo signaling, decreasing LATS1/2 phosphorylation, thus allowing YAP/TAZ/TEAD to transcribe genes related to growth, survival, and cell cycle progression. When BMI1 is inhibited pharmacologically or genetically, LATS1/2 are phosphorylated, leading to YAP degradation and diminishing the transcription of YAP/TAZ/TEAD target genes. (E) Rh28 and Rh30 cells were transiently transfected with pooled siRNAs against LATS1, LATS2, or both, with a nontargeting pool as a control (siCtl). Western blot shows protein levels of LATS1, LATS2, YAP, with GAPDH as a loading control. (F) Dose–response curve of PTC‐028 ranging from 10^−11^ m–10^−5^ m, using transiently transfected siCtl, siLATS1, siLATS2, siLATS1/2 cells from (E). (G) Table summary of PTC‐028 IC50s from (F). (H) Western blot representing stably lentivirus‐transduced cell lines Rh28/Rh30 pGAMA‐Empty (Empty) and Rh28/Rh30 pGAMA‐YAP. pGAMA‐YAP contains mCherry‐tagged YAP (mCh‐YAP) which runs at a higher molecular weight (≈ 85 kDa) than endogenous YAP (≈ 65 kDa). GAPDH as a loading control. (I) Dose–response curve of PTC‐028 ranging from 10^−11^ m to 10^−5^ m, using stable cell lines Rh28‐Empty, Rh28 + YAP, Rh30‐Empty, and Rh30 + YAP from (H). (J) Table summary of PTC‐028 IC50s from (I). Results are representative of at least three independent biological replicates (*n* = 3).

We initially explored the effects of BMI1 inhibition on canonical Hippo signaling. Normally, YAP/TAZ binds TEAD and YAP/TAZ/TEAD complexes influence groups of genes implicated in cell cycle progression and growth [55]. MST1 phosphorylates and activates LATS1/2, which in turn phosphorylates YAP/TAZ, leading to YAP/TAZ degradation and exclusion from the nucleus, with subsequent reduction in the amount of YAP/TAZ/TEAD complexes capable of transcriptional activation [55]. Upon treatment with PTC‐028, we observed that LATS1/2 phosphorylation increases, and YAP levels decrease (Fig. 6A), indicating that the Hippo pathway is activated when BMI1 is inhibited. However, there is no change in total LATS1/2 levels or MST1 phosphorylation (Fig. S4B, C), suggesting a possible alternative mechanism for the increase in LATS1/2 phosphorylation. We depleted BMI1 using siRNAs and similarly observed an increase in LATS1/2 phosphorylation and a decrease in YAP protein expression (Fig. 6B). BMI1 inhibition appears to promote Hippo pathway activation through LATS1 phosphorylation. We then looked further downstream at several canonical YAP/TAZ targets. We chose several canonical YAP/TAZ targets, AXL, CYR61 (CCN1), and CTGF (CCN2), which are involved in processes such as cellular proliferation, cell cycle progression, and cell migration/invasion [55, 56, 57, 58]. We found that levels of all these proteins decrease with PTC‐028 addition (Fig. 6C). Our current model (Fig. 6D) summarizes these data, which suggest that BMI1 promotes tumor cell growth by inhibiting LATS1/2 phosphorylation and allowing YAP/TAZ/TEAD to transcribe canonical target genes (such as AXL, CYR61, and CTGF). When BMI1 is lost, either by genetic knockdown or PTC‐028 treatment, Hippo is activated, LATS1/2 remain phosphorylated and YAP is subsequently degraded (Fig. 6D).

Next, we sought to demonstrate that BMI1 promotes cell proliferation through the inhibition of Hippo signaling; we took two approaches to rescue the phenotype of BMI1 inhibition. First, in Rh28 and Rh30, we knocked down LATS1, LATS2, or both LATS1/2 by siRNA transfection (Fig. 6E) and showed that YAP levels increase when LATS1 or both LATS1/2 are knocked down. Then, we treated cells transfected with siCtl, siLATS1, siLATS2, and siLATS1/2 with PTC‐028 at a 7‐log range of doses (10^‐11^ m–10^‐5^ m) to recalculate IC50s (Fig. 6F). We found that knockdown of LATS1, LATS2, and LATS1/2 increased PTC‐028 IC50s approximately twofold to fourfold compared with control (Fig. 6G). In our second approach, we overexpressed YAP directly by transducing Rh28 and Rh30 cells with lentiviral particles containing pGAMA‐Empty (empty vector) or pGAMA‐YAP (YAP tagged with mCherry), and confirmed overexpression by western blot (Fig. 6H). We treated Rh28/Rh30 pGAMA‐empty and pGAMA‐YAP cell lines with a 7‐log range of doses (10^−11^ m–10^−5^ m) and calculated IC50s (Fig. 6I,J). The IC50s were approximately twofold to threefold higher in YAP‐overexpressing cells (Fig. 6J), suggesting a link between BMI1 and LATS1/2‐YAP signaling within the Hippo pathway. Collectively, these data demonstrate that BMI1 influences cell proliferation by negatively regulating Hippo signaling and that the effects of BMI1 inhibition can be partly reversed through the inhibition of LATS1/2 and the upregulation of YAP.

## 4 DISCUSSION

Our understanding of, and hence optimal treatment for ARMS, remains inadequate. Motivated by a growing understanding that PAX3‐FOXO1 fusion proteins interact with diverse epigenetic complexes, including BRD4 [11, 12] and CHD4 [13], we hypothesized that BMI1 would contribute to ARMS aggression and that inhibiting this protein could potentially confer therapeutic benefit. Importantly, while studies suggest that BMI1 inhibition is a downstream effect of PTC‐028 [27], our studies show that genetic depletion of BMI1 using multiple independent siRNAs/shRNAs diminishes proliferation (Fig. 2). Moreover, we find that pharmacologic disruption using PTC‐209, which inhibits effective translation of *BMI1* mRNA [48], decreases ARMS cellular viability significantly (Fig. 3). We provide evidence that BMI1 inhibition diminishes cell cycle progression and increases apoptosis (Fig. 4).

In the *in vivo* setting, we show that single‐agent treatment significantly decreases, though does not abrogate, ARMS growth (Fig. 5). Notably, while PTC‐028 displays better *in vivo* characteristics than PTC‐209, PTC‐028 is still an early generation inhibitor. PTC‐596 is the clinical analog of PTC‐028 that has recently entered into clinical trials for patients with advanced solid malignancies [59]. A1016 is an additional BMI1 inhibitor related to PTC‐596 and has shown similar positive results in glioblastoma [27]. Future investigations will investigate the impact of these newer generation inhibitors on ARMS. Recently, investigators showed that the combination of PTC‐596 and standard chemotherapy (gemcitabine and nab‐paclitaxel) resulted in regressions in multiple aggressive pancreatic cancer models and, importantly, was well‐tolerated [60]. Based on such studies, we speculate that combining BMI1 inhibition with standard‐of‐care chemotherapeutic regimens in RMS may both be well‐tolerated and result in greater inhibition of tumor growth, though further studies are needed to investigate this hypothesis.

While the current study delineates the impact of BMI1 on cell cycle progression and evasion of apoptosis, BMI1 has been implicated in multiple hallmarks of cancer, including DNA repair and self‐renewal [50]. In melanoma, *BMI1* expression was shown to be correlated with an invasive signature and to promote multiple aspects of melanoma metastasis, including anoikis, invasion, migration, and chemoresistance [61]. Might BMI1 contribute to metastatic dissemination in ARMS and could disruption of its function impede metastatic dissemination? Finally, while our studies focused on ARMS, we find that *BMI1* is broadly expressed in multiple pediatric and adult sarcomas (Fig. 1), raising the possibility that BMI1 may shape the initiation, maintenance, and progression of diverse sarcoma histotypes. To facilitate such studies, it would be of substantial interest to investigate the impact of BMI1 overexpression and deletion on various genetically engineered sarcoma mouse models (GEMMs).

In addition to proposing a role for BMI1 in ARMS, our studies also reveal the influence of BMI1 on Hippo signaling and raise further mechanistic questions. For example, we find that inhibition of BMI1 results in increased levels of LATS1/2 phosphorylation at Thr1079/Thr1041, which is associated with LATS1/2 activation [62]. However, inhibiting BMI1 does not appear to influence either the expression or phosphorylation of MST1, which lies upstream of LATS1 (Fig. S4A). It is possible that BMI1 epigenetically represses an unidentified kinase of LATS1/2, or perhaps BMI1 engages with LATS1/2 through protein–protein interactions (Fig. 6D). This would be especially novel, considering the canonical role of BMI1 almost exclusively acting through epigenetic mechanisms. More investigation will be necessary to define the upstream mechanism of action by which BMI1 influences Hippo signaling. Looking downstream, we find that YAP protein levels decrease upon BMI1 inhibition, and several downstream canonical YAP/TAZ targets (AXL, CYR61, and CTGF) all decrease at the protein level (Fig. 6C). This is consistent with our hypothesis that BMI1 suppresses Hippo pathway activation. [50]. We additionally rescued the effects of BMI1 inhibition by PTC‐028 by knocking down LATS1, LATS2, and LATS1/2, confirming that YAP levels increase and negate the antiproliferative effects of BMI1 inhibition (Fig. 6E–G). We performed a complementary experiment wherein we overexpressed YAP directly and found similar results (Fig. 6H–J), further confirming the significance of Hippo signaling in ARMS through BMI1. Interestingly, in undifferentiated pleomorphic sarcomas, there is evidence for the deregulation of the Hippo pathway and subsequent activation of YAP/TAZ [63]. It is intriguing to posit a broad role for BMI1 involvement in the Hippo pathway across sarcomas and to speculate that BMI1 inhibition may provide a method of activating the Hippo pathway in these malignancies.

In conjunction with further dissection of BMI1‐Hippo signaling, it will be important to define the full repertoire of genes influenced by BMI1 using both RNA‐seq and ChIP‐seq approaches, and to see how BMI1‐influenced genes converge and diverge from other malignancies by analyzing target gene expression states [27, 51, 64]. Furthermore, it will be of substantial interest to determine whether BMI1 acts through its canonical role as a member of the PRC1 complex, or by associating with other complexes to control gene expression in ARMS. There are six canonical PRC1 complexes (and even more noncanonical), each with a different PCGF (BMI1 is PCGF4). It would be interesting to determine whether the inhibition of other PRC1 complexes would have similar effects to targeting BMI1 [65]. Perhaps combining inhibitors of different PRC1 complexes could have a synergistic effect, as it is possible that PRC1 groups could compensate for another if one or more is lost. Moreover, what effects does BMI1 inhibition have on global chromatin changes? Additional ChIP‐seq experiments investigating where BMI1/PRC1 localizes in ARMS, then further exploring the impact of BMI1 inhibition on histone repressive marks such as H2AK119Ub and H3K27me3, along with active marks like H3K27ac, will help clarify the molecular mechanisms by which BMI1 influences the malignant phenotype.

## 5 Conclusions

Our studies propose a novel role for BMI1 signaling in ARMS, connect BMI1 the Hippo pathway, and raise additional questions with regard to BMI1 function and signaling. They provide a strong foundation for investigating the utility of BMI1 inhibition in ARMS and should spur further investigations of BMI1 and other PRC1/2 proteins as potential dependencies in RMS and other sarcomas.

## Conflict of interest

Robert W. Schnepp reports employment at Janssen R&D. There are no other conflicts of interest.

## Author contributions

CES, KMO, CML, KAH, and RWS contributed to conception and design. CES and RWS involved in development of methodology. CES, SP, SMC, SKC, DC, DM, JP, SP, and RWS contributed to acquisition of data. CES, KSR, and RWS analyzed and interpreted the data (biostatistics, statistical analysis, interpretation of clinical data, and genomic datasets). CES, KAH, and RWS wrote, reviewed, and/or revised the manuscript. KMO, CML, JMM, KAH, and RWS involved in administrative, technical, or material support. KAH and RWS supervised the study.

## Supporting information


**Fig. S1.** *BMI1* is highly expressed in rhabdomyosarcoma.
**Fig. S2.** Pharmacologic inhibition of BMI1 decreases cell proliferation *in vitro*.
**Fig. S3.** Targeting BMI1 decreases cell cycle progression and increases apoptosis in FP‐RMS.
**Fig. S4.** BMI1 negatively influences Hippo signaling.

Table S1. List of antibodies, sources and dilutions used in all western blot assays.
