# Supplementary material for: Epigenetic regulator BMI1 promotes alveolar rhabdomyosarcoma proliferation and constitutes a novel therapeutic target
Source: Mol Oncol. 2021 Mar 27;15(8):2156–71. doi: 10.1002/1878-0261.12914 (PMC8333775; doi:10.1002/1878-0261.12914)

Supplementary Figure S1

A

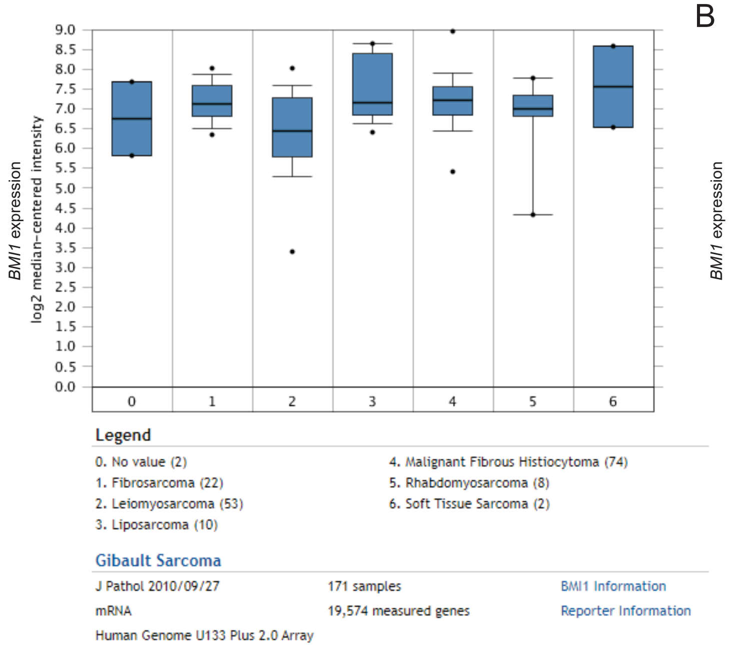

B

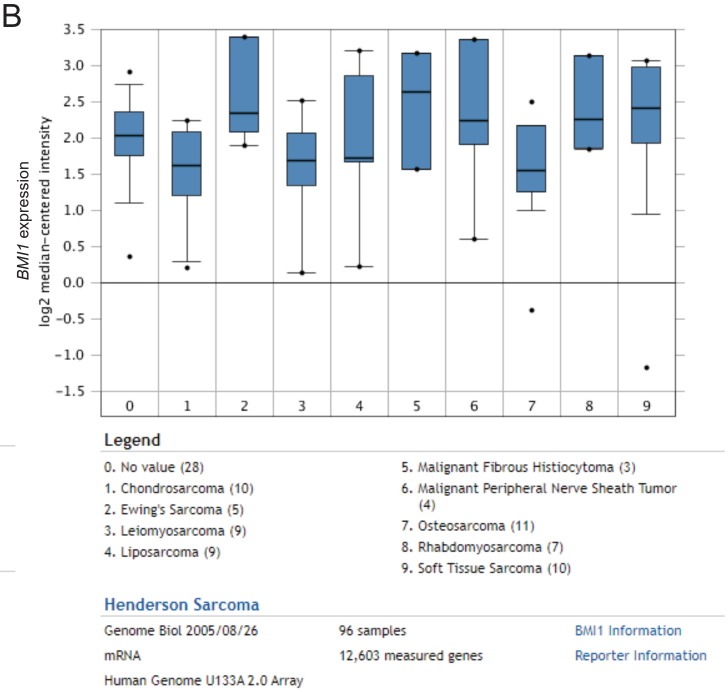

C

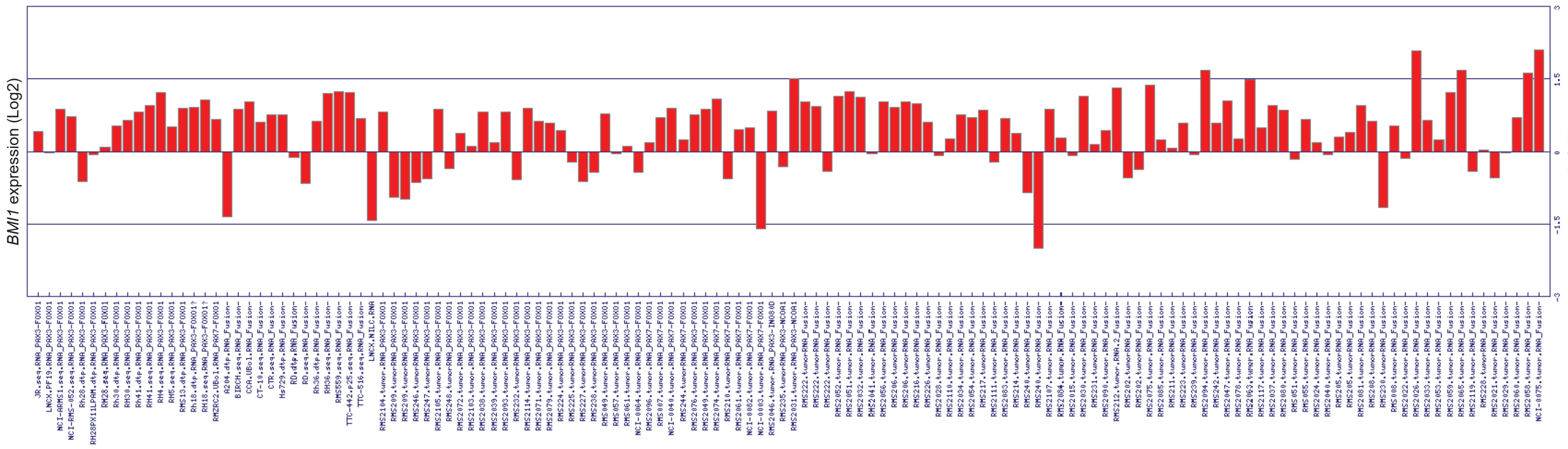

Supplementary Figure S2

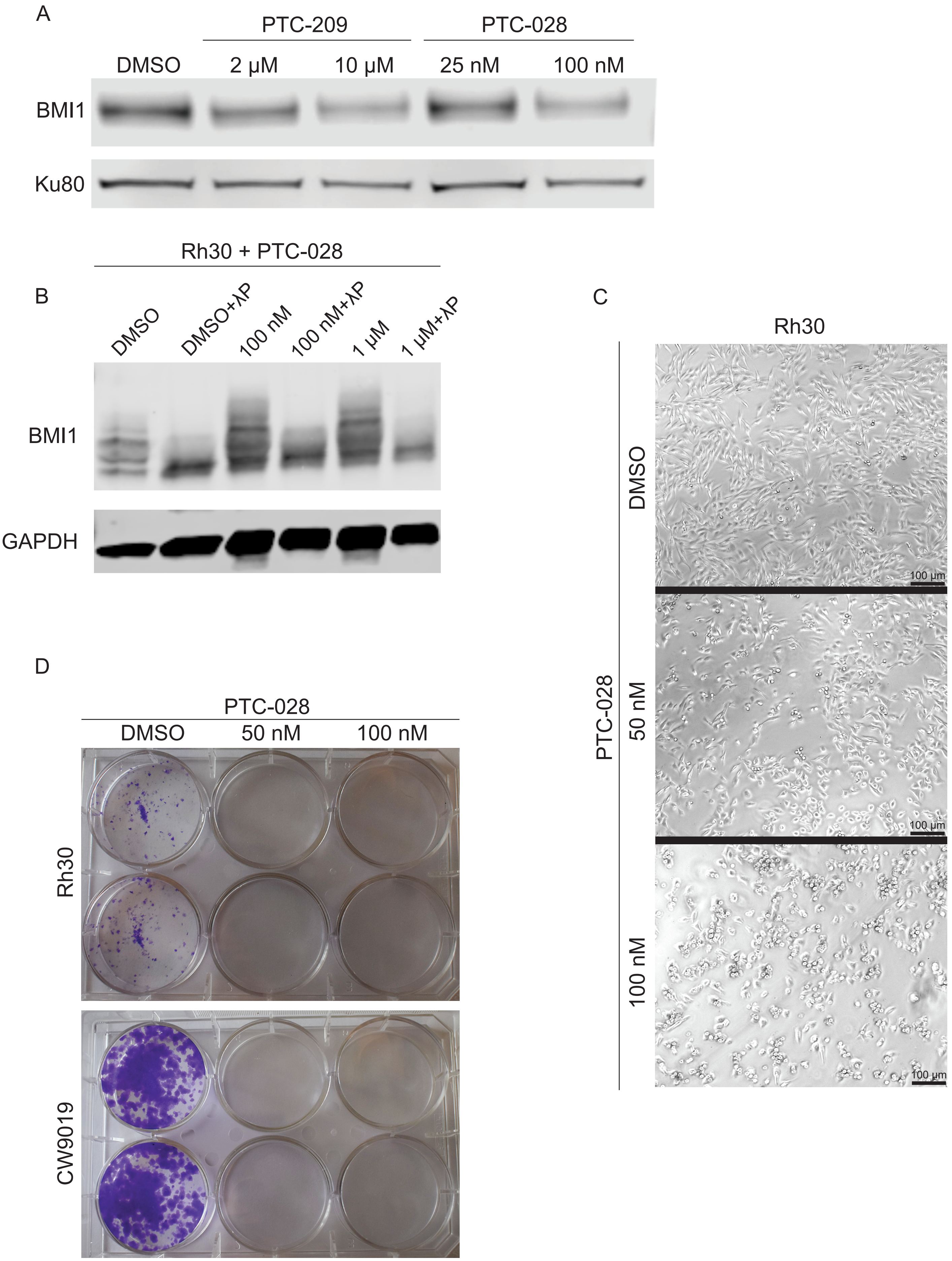

Supplementary Figure S3

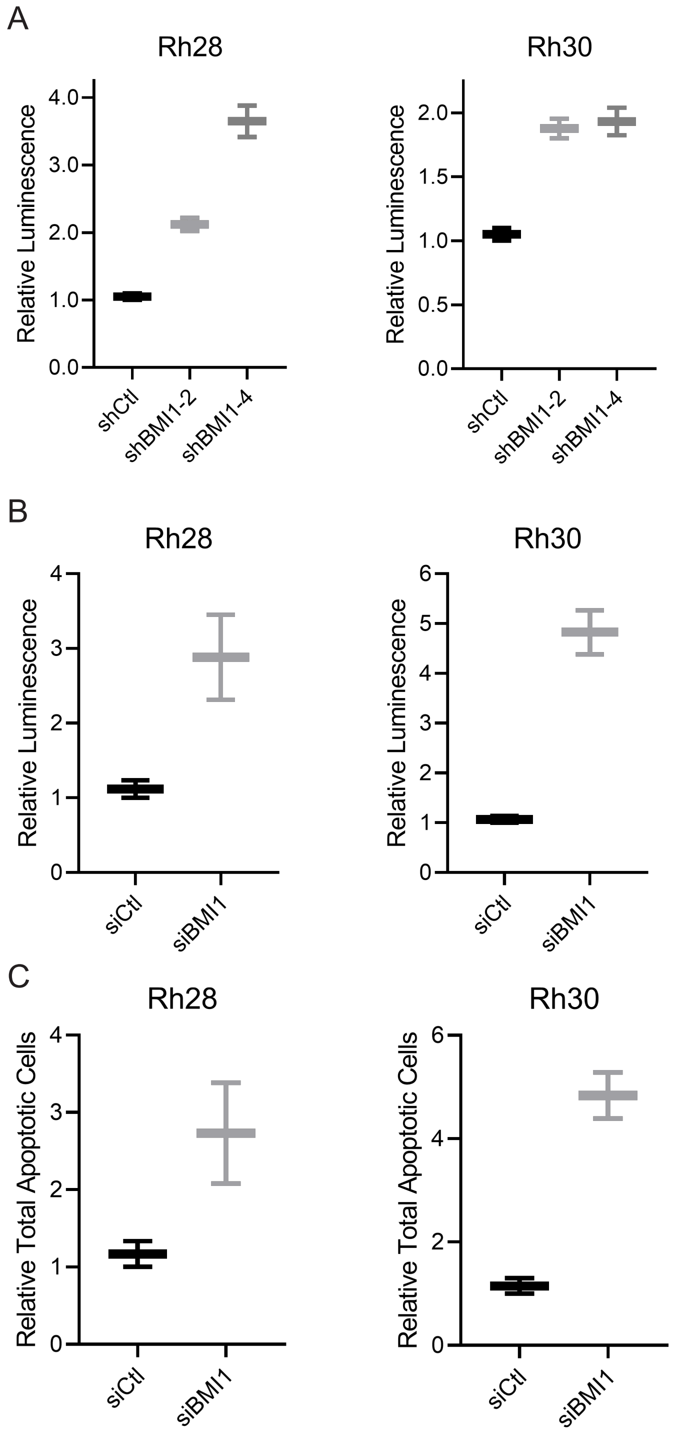

Supplementary Figure S4

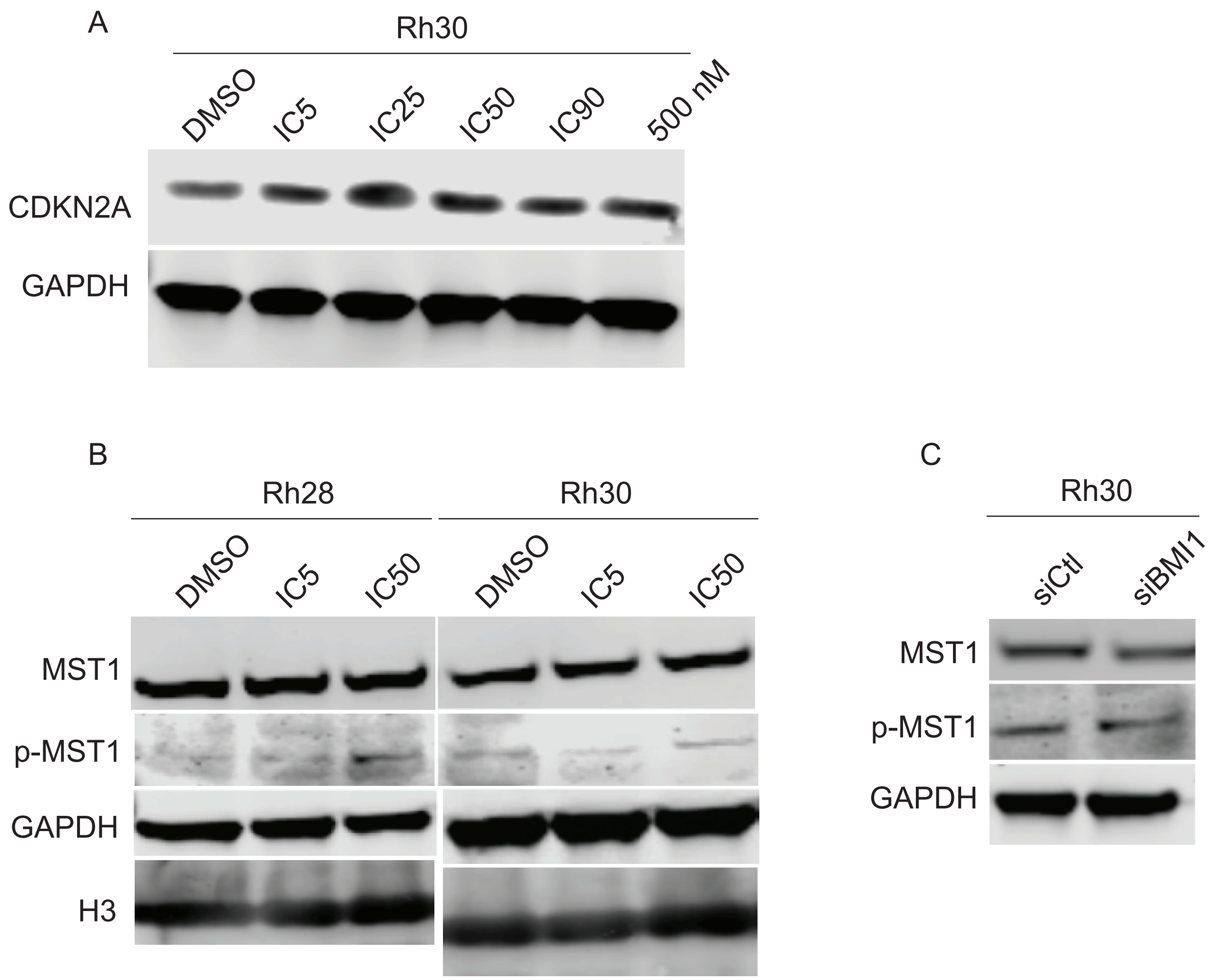

Supplement: Supplementary file 1 — Fig. S1. BMI1 is highly expressed in rhabdomyosarcoma. Fig. S2. Pharmacologic inhibition of BMI1 decreases cell proliferation in vitro. Fig. S3. Targeting BMI1 decreases cell cycle progression and increases apoptosis in FP‐RMS. Fig. S4. BMI1 negatively influences Hippo signaling. [file MOL2-15-2156-s001.pdf]
