# Supplementary material for: Epigenetic regulator BMI1 promotes alveolar rhabdomyosarcoma proliferation and constitutes a novel therapeutic target
Source: Mol Oncol. 2021 Mar 27;15(8):2156–71. doi: 10.1002/1878-0261.12914 (PMC8333775; doi:10.1002/1878-0261.12914)
Supplement: Supplementary file 2 — Table S1. List of antibodies, sources and dilutions used in all western blot assays. [file MOL2-15-2156-s002.docx]

**Supplementary Table 1.** Antibodies used to produce the western blot data shown in Figures 1, 2, 4, 5, 6, and supplementary figures S2 and S4. CST = Cell Signaling Technologies.

| **Antibody Target** | **Vendor** | **Catalog #** | **Species** | **Clonality** | **Dilution** |
| --- | --- | --- | --- | --- | --- |
| BMI1 | CST | 6964 | Rabbit | mAb | 1:1000 |
| Ku80 | CST | 2180 | Rabbit | mAb | 1:5000 |
| Cleaved PARP | CST | 5625 | Rabbit | mAb | 1:1000 |
| β-Actin | CST | 4967 | Rabbit | pAb | 1:1000 |
| GAPDH | CST | 5174 | Rabbit | mAb | 1:5000 |
| MST1 | CST | 14946 | Rabbit | mAb | 1:1000 |
| p-MST1/2 (Thr183)/(Thr180) | CST | 49332 | Rabbit | mAb | 1:500 |
| LATS1 | CST | 3477 | Rabbit | mAb | 1:1000 |
| p-LATS1/2 (Thr1079)/(Thr1041) | CST | 8654 | Rabbit | mAb | 1:500 |
| YAP/TAZ | CST | 8418 | Rabbit | mAb | 1:500 |
| AXL | CST | 8661 | Rabbit | mAb | 1:500 |
| CYR61 | CST | 14479 | Rabbit | mAb | 1:500 |
| CTGF | CST | 86641 | Rabbit | mAb | 1:500 |
| CDKN2A (p16-INK4A) | Proteintech | 10883-1-AP | Rabbit | pAb | 1:1000 |
| Histone H3 | Abcam | ab1791 | Rabbit | pAb | 1:1000 |
